# A health union in support of European and national health solidarity

**DOI:** 10.1016/j.lanepe.2024.101051

**Published:** 2024-08-29

**Authors:** Frank Vandenbroucke

**Affiliations:** Vice-Prime Minister and Minister of Health and Social Affairs, Belgium

What should be the ambition of a European Health Union? This was the fundamental question put forward by the Belgian government during its Presidency of the EU, a Presidency which ended in June 2024 and aimed at defining the EU's strategic agenda on health for the next five years.^1^

Ursula von der Leyen coined the expression ‘a European Health Union’ in 2020, building on initiatives taken in response to COVID-19 and drawing lessons from that experience. Notwithstanding a broad mission statement, the measures taken under the umbrella of the Health Union mostly focused on preparedness and response to emergencies, and less on the overall resilience of national public health systems. Yet, the pandemic demonstrated that accessible and high-quality health systems and a healthy population enhance resilience to emergencies. A true European Health Union should not only pursue an ambitious agenda of European-level risk-sharing and collective action (notably to tackle medicines shortages) but take the resilience of public health systems fully on board in its objectives, so the Belgian government argued.

This explains why we (i.e. the Belgian government) not only rallied support, together with the European Commission, for measures to tackle medicines shortages (ranging from the organisation of an intra-European solidarity mechanism to address shortages in singular member states, to the launching of a Critical Medicines Alliance to overcome critical dependencies in the production of basic medicines), but also initiated a debate on health workforce shortages and the role EU legislation plays in this—next to other topics which we wanted on the next five years’ agenda. This also explains why we argued that well-organized healthcare spending is a social investment that comes with a return; it should not fall victim of a one-sided emphasis on fiscal consolidation in the new budgetary rules of the EU.

European public health systems are premised on principles of risk-sharing and redistribution to ensure universal access to healthcare. Therefore, a Health Union is about a shared commitment to these solidarity principles. A Health Union should also mandate or encourage its member countries to enhance prevention and other public health policies to create a more equal baseline of public health before inequalities arise.^2^ The social gradient of preventable risk factors such as smoking and drinking is well known, and prevention is an egalitarian policy *par excellence*. The European internal market creates a unique opportunity for collective action on tobacco, alcohol, gambling and healthy food. The EU's action on tobacco and alcohol has stalled over the last few years: it should be relaunched, a point made during our Presidency. I was therefore pleased to see that Ursula von der Leyen included prevention in the Policial Guidelines for her second term of office.^3^

The European Union should also use its ‘market-shaping’ power to pursue the universal accessibility of new medicines to all Europeans, in large and small, rich and poor member states alike—which is not the logic of the pharmaceuticals market when left to its own. Universal access is one of the objectives of the Commission's pharmaceutical package. This is hotly debated, but a Health Union should indeed pursue universal access to medicines and apply the EU's market authorisation powers to that end. From a citizen's perspective, this is a litmuss test for a solidaristic European Health Union.

Undoubtedly, a European Health Union cannot and should not replace national health care systems and public health policies: its mission is to provide systemic support to national systems and policies, whilst maintaining subsidiarity as an organizing principle.^4^ But subsidiarity must not become an excuse for inaction. Questions of subsidiarity exercise first and foremost national policymakers nervous about their own competences. Citizens are worried about shortages of medicines, unaffordable new drugs, staff shortages: they have to see that the EU comes to their aid through tangible collective action.

## Contributors

Frank Vandenbroucke as the sole author of this comment contributed to all aspects of the manuscript.

## Declaration of interests

FV has no competing interests to declare.
